# The Impact of Omnivorous Feeding on Antioxidant Capacity, Gut Microbiota, and the Relationship Between Gut Microbiota and Meat Quality in Xinghua and White Recessive Rock Chickens

**DOI:** 10.3390/ani16213392

**Published:** 2026-10-27

**Authors:** Jing-E Ma, Jie Liang, Bahareldin Ali Abdalla Gibril, Jishang Gong, Yuanfei Li, Qiao Xu, Xuewen Chai, Wen Luo, Jiguo Xu

**Affiliations:** 1 Jiangxi Provincial Key Laboratory of Poultry Genetic Improvement, Institute of Biological Technology, Nanchang Normal University, Nanchang 330032, China; majinge@ncnu.edu.cn (J.-E.M.); gongjishang@126.com (J.G.);; 2 State Key Laboratory of Livestock and Poultry Breeding, and Lingnan Guangdong Laboratory of Agriculture, South China Agricultural University, Guangzhou 510642, China; 3 Department of Animal Genetics, Breeding and Reproduction, College of Animal Science, South China Agricultural University, Guangzhou 510642, China; 4 Guangdong Provincial Key Laboratory of Agro-Animal Genomics and Molecular Breeding, and Key Laboratory of Chicken Genetics, Breeding and Reproduction, Ministry of Agriculture and Rural Affairs, South China Agricultural University, Guangzhou 510642, China

**Keywords:** gut microbiota, meat quality, oxidative stress, intestinal morphology, correlation analysis

## Abstract

The impacts of omnivorous feeding on intestinal morphology and microbial community structure remain a critical area of research in poultry science, uncovering potential biological mechanisms regulating growth and development. This study investigated how feeding regimes modulate antioxidant enzyme activities, intestinal morphological structure, gut microbiota, and the relationship between gut microbiota and meat quality. We conducted a comprehensive analysis integrating antioxidant enzyme activities, intestinal histomorphology, and 16S rRNA gene sequencing. Correlation analyses were performed to identify key microbial taxa influencing meat quality. Omnivorous feeding significantly altered the activities of certain antioxidant enzymes in the serum and selected intestinal tissues of both White Recessive Rock and Xinghua chickens. Omnivorous feeding significantly reduced the VH/CD ratio in specific intestinal segments. Microbial analysis revealed that omnivorous feeding modulated the relative abundances of *Bacillus*, *Enterococcus*, *Escherichia-Shigella*, and *Romboutsia.* These shifts promoted metabolic functions, including carbohydrate, amino acid, and energy metabolism. Crucially, correlation analysis identified *Faecalibacterium*, *Bacillus*, *Romboutsia*, *Streptococcus*, and unclassified *UCG_010* as key genera significantly associated with meat quality traits. The findings suggest that omnivorous feeding shows the potential to improve poultry health and meat quality.

## Simple Summary

This study examined the effects of omnivorous feeding on antioxidant capacity, intestinal morphological structure, gut microbiota, and meat quality in Xinghua and White Recessive Rock chickens. The results show that omnivorous feeding significantly altered the activities of antioxidant enzymes in serum or specific intestinal tissues and decreased the villus height-to-crypt depth (VH/CD) ratio in specific intestinal segments. Microbial analysis revealed that this feeding regime reshaped the gut microbiota structure and modulated beneficial bacteria such as *Lactobacillus* and *Bifidobacterium* while altering related metabolic pathways. Furthermore, correlation analysis demonstrated that key genera, such as *Bacillus*, were significantly associated with meat quality traits, including pH, meat color, and shear force. Collectively, these findings provide novel insights into the regulatory mechanisms linking omnivorous feeding, gut microbiota modulation, and meat quality traits. Theoretically, this study enriches the understanding of host–microbiome interactions under distinct dietary regimes. Practically, the identification of breed-specific responses offers valuable guidance for designing precision feeding protocols. These insights are critical for optimizing feed efficiency and meat quality, thereby promoting the sustainable utilization of omnivorous feed resources in animal production.

## 1. Introduction

A comprehensive characterization of the gut microbiota in healthy chickens across distinct developmental stages and intestinal segments is essential for understanding how it supports normal metabolism and growth. The gut microbiota is primarily composed of bacteria, viruses, fungi, protozoa and archaea, which coexist with the animal body [1]. The gut genome, often referred to as the acquired “second genome” of the host, exhibits extensive genetic and metabolic diversity [2]. The composition of the microbiota varies significantly at different developmental stages [3]. These different microbiota compositions interact with the host mainly through their metabolites and metagenomes, exerting various effects on the host [4]. Five bacterial strains in broilers are closely associated with intestinal mucosal immune function [5]. The gut microbiota can enhance the feed utilization rate of animals. When poultry are fed identical amounts of feed, significant variations in gut microbial composition are observed, which correlate with differences in the feed conversion rate [6]. Previous studies have shown a correlation between gut microbial diversity and feed efficiency [7]. Chickens with a higher feed utilization rate exhibit a greater abundance of Bacteroides and Akkermansia in the cecum. The cecum of chickens with a high feed utilization rate is also enriched with microbial communities dominated by Lactobacillus [8].

The gut microbiota is highly susceptible to dietary perturbations. Studies have shown that supplementation with probiotics such as Bacillus licheniformis and Saccharomyces cerevisiae significantly increases serum levels of growth hormone, insulin-like growth factor 1, and insulin [9]. Moreover, Lactobacillus plantarum supplementation has been found to promote growth and development [10]. Alterations in the gut microbiota of broilers affect the expression of some amino acid transporters and ion transporters, which consequently have an impact on their growth and development [11]. How omnivorous feeding influences intestinal morphology and microbial community structure is still not well characterized.

Antioxidant capacity represents the cumulative action of all antioxidants present in a sample, providing an integrated parameter of the body’s overall defense against oxidative stress [12]. Several factors, including breed, age, and diet, have been shown to influence antioxidant capacity [13,14,15]. Local breeds like XH chickens exhibit superior meat quality due to a higher inosine monophosphate content, finer muscle fibers, and lower shear force than commercial broilers [16]. However, the molecular mechanisms underlying these breed differences remain poorly understood, particularly in the context of dietary interventions.

The present study utilized two broiler breeds with distinct growth and development rates, White Recessive Rock and Xinghua chickens, as experimental models, as mentioned in a previous study [16]. It aimed to elucidate the impacts of omnivorous feeding on intestinal morphology and microbial community structure, ultimately uncovering potential biological mechanisms regulating growth and development. The research further explored whether modern feeding practices using formulated diets offer superior safety and comprehensive nutrition compared to omnivorous feeding while examining the potential impacts of omnivorous feeding on chicken health and production performance. The findings are anticipated to establish a scientific foundation for the genetic improvement of broiler strains in future breeding programs.

## 2. Materials and Methods

### 2.1. Ethics Statement

All animal experiments were carried out in compliance with protocols approved by the Institutional Animal Care and Use Committee of South China Agricultural University (approval number: SCAU#2024A004) on 23 February 2023. All efforts were made to minimize animal suffering.

### 2.2. Sample Collection

A total of 120 one-day-old chicks, 60 Xinghua chickens (local breed; Zhicheng Poultry Breeding Co., Ltd., Zhaoqing, China), and 60 White Recessive Rock chickens (commercial broiler; Guangzhou Jiangfeng Seed Industry and Technology Co., Ltd., Guangzhou, China) were selected and housed under standardized environmental conditions with unrestricted access to water. Chicks were stratified by breed and randomly assigned to four experimental groups (*n* = 30 per group): Xinghua chickens fed an omnivorous diet (XH_EG), Xinghua chickens fed a standard diet (XH_CG), White Recessive Rock chickens fed an omnivorous diet (WRR_EG), and White Recessive Rock chickens fed a standard diet (WRR_CG). The control groups received a nutritionally complete commercial broiler diet (Chia Tai Group, Beijing, China). The experimental groups were fed an omnivorous diet designed to mimic natural foraging, consisting of 50% cooked rice, 30% rice husks, and 20% fresh vegetables (leafy greens, carrots, and cabbage) (Table S1). The birds were fed twice daily (08:00 and 16:00) for 8 weeks. The details of the animals and experimental design are described in a previous study [16].

Slaughtering and sampling were conducted at 5 and 8 weeks of age. Blood samples were collected from these chickens prior to slaughter; then, they were left to stand at room temperature for 1 h, centrifuged at 3000 r/min for 10 min to separate the serum, and subsequently stored at −80 °C. For 16S rRNA sequencing, intestinal contents from the duodenum, jejunum, ileum, cecum, and fecal samples were collected from all chickens and stored in sterile cryopreservation tubes at −80 °C. For oxidative stress determination, tissue samples from the duodenum, jejunum, and ileum were also collected and stored in sterile cryopreservation tubes at −80 °C. Additionally, approximately 2 cm segments of the duodenum, jejunum, and ileum were collected from each chicken, fixed in 4% paraformaldehyde solution, and processed for histological sectioning and hematoxylin–eosin (H&E) staining. The sample size for histological, biochemical, and meat quality analyses was *n* = 6 per group. The microbiota and meat quality data were obtained from the same individual birds. Specifically, after recording the meat quality traits (e.g., pH, color, and shear force) [16], the intestinal contents were immediately collected from the corresponding carcass to ensure a precise one-to-one pairing for the correlation analysis.

### 2.3. Determination of Antioxidant Parameters

Antioxidant parameters were determined in the serum and duodenum, jejunum, and ileum tissues of the chickens. Total protein concentration was measured using a BCA Protein Assay Kit (Beyotime, Shanghai, China). The activities of various enzymes were analyzed using the assay kits (Nanjing Jiancheng Bioengineering Institute, Nanjing, China) according to the manufacturer’s instructions. Total superoxide dismutase (SOD) activity was measured using the WST-1 method. Glutathione S-transferase (GSH-ST) activity, lactate dehydrogenase (LD) activity, and myeloperoxidase (MPO) activity were determined using colorimetric methods. Catalase (CAT) activity was assayed using the ammonium molybdate method. Diamine oxidase (DAO) activity was determined using a spectrophotometric method. Reactive oxygen species (ROS) levels were determined using a chemiluminescence assay.

### 2.4. Hematoxylin and Eosin (H&E) Staining

Intestinal segments from the duodenum, jejunum, and ileum were subjected to H&E staining. Briefly, the tissue samples were fixed in 4% paraformaldehyde solution for at least 24 h. The tissues were then dehydrated through a graded ethanol series, embedded in paraffin, and trimmed into blocks after cooling on ice. The blocks were sectioned at 4 μm using a microtome. The sections were stained using an H&E staining kit. Histological observations and photography were performed using a Leica DM2500 microscope (Leica, Wetzlar, Germany). The villus height (VH) and crypt depth (CD) were measured using the Image-Pro Plus image analysis system. Three microscopic fields per section were selected randomly by moving the slide along the x-axis. The observer was blinded to the treatment groups during measurement. For each chicken, 15 well-oriented, intact villi and their corresponding crypts were measured to calculate the average values of the VH, CD, and villus height-to-crypt depth ratio (VH/CD). These individual bird means were used for statistical analysis.

### 2.5. DNA Extraction and Purification

Microbial DNA was extracted from intestinal contents and fecal samples using a TGuide S96 Magnetic Stool DNA Kit (Tiangen Biotech, Beijing, China) according to the manufacturer’s instructions. DNA concentration was quantified using a Qubit fluorometer (Thermo Fisher, San Diego, CA, USA). The V3-V4 hypervariable regions of the 16S rRNA gene were amplified using a two-step PCR protocol. In the first-round PCR, locus-specific primers (Table S2) containing partial Illumina adapter overhangs were used to amplify the target region. *PCR* amplification was performed in a 20 μL reaction mixture (Table S3). The cycling conditions are described in Table S4. The purified first-round product was then used as the template for the second-round PCR, incorporating sample-specific dual indices and full Illumina sequencing adapters for multiplexed sequencing. The PCR reaction mixture and thermal cycling conditions are detailed in Tables S5 and S6. The target amplicons were subsequently purified using a Monarch DNA Gel Extraction Kit. Following library construction, library quality was assessed, and qualified libraries were sequenced on the Illumina NovaSeq 6000 platform (San Diego, CA, USA).

### 2.6. 16S rRNA Data Analysis

Raw sequencing reads were quality-filtered using Trimmomatic v0.33. The software Cutadapt 1.9.1 was used to identify and remove primer sequences, yielding processed reads. QIIME2 (Dada2, version 2020.6) was utilized for denoising, merging paired-end sequences, and removing chimeric sequences to obtain the final set of high-quality, non-chimeric reads. Based on the processed Amplicon Sequence Variants (ASVs), both alpha and beta diversity analyses were performed. Alpha diversity was assessed using multiple metrics, including Chao1, Ace, Shannon, Simpson, Coverage, and PD_whole_tree. Beta diversity analysis was primarily conducted using the phyloseq package to perform Principal Coordinates Analysis (PCoA). Linear discriminant analysis (LDA) effect size (LEfSe) analysis was performed using the Python (Version 1.0.8) LEfse package to identify differentially abundant features across groups. Finally, PICRUSt2 was employed to predict KEGG functional profiles and metabolic pathways based on the SILVA v138 reference database.

### 2.7. Data Analysis

All data are expressed as the mean ± standard deviation (SD). Analysis of variance (ANOVA) was performed using SPSS 26.0 for group comparisons. Post hoc multiple comparisons were conducted using the Least Significant Difference (LSD) test or Duncan’s test. A value of *p* < 0.05 was considered statistically significant. Due to the multiple comparisons involved in the microbiome analysis and correlation tests, *p*-values were adjusted using the Benjamini–Hochberg (BH) false discovery rate (FDR) method.

## 3. Results

### 3.1. Effects of Omnivorous Feeding and Breed Differences on Antioxidant Capacity

Six parameters were assessed in the serum, duodenum, jejunum, and ileum to evaluate the effect of omnivorous feeding on antioxidant capacity, including CAT, DAO, GSH-ST, LD, MPO, and SOD. Additionally, ROS levels were evaluated in the intestinal tract. Breed comparison analysis revealed that SOD activity in the duodenum (*p* = 0.042), DAO activity in the jejunum (*p* = 0.025), and ROS levels in the ileum of Xinghua chickens were significantly lower than those of White Recessive Rock chickens (*p* = 0.006). Conversely, SOD activity in the ileum of Xinghua chickens was significantly higher (*p* = 0.045).

**Figure 1:**
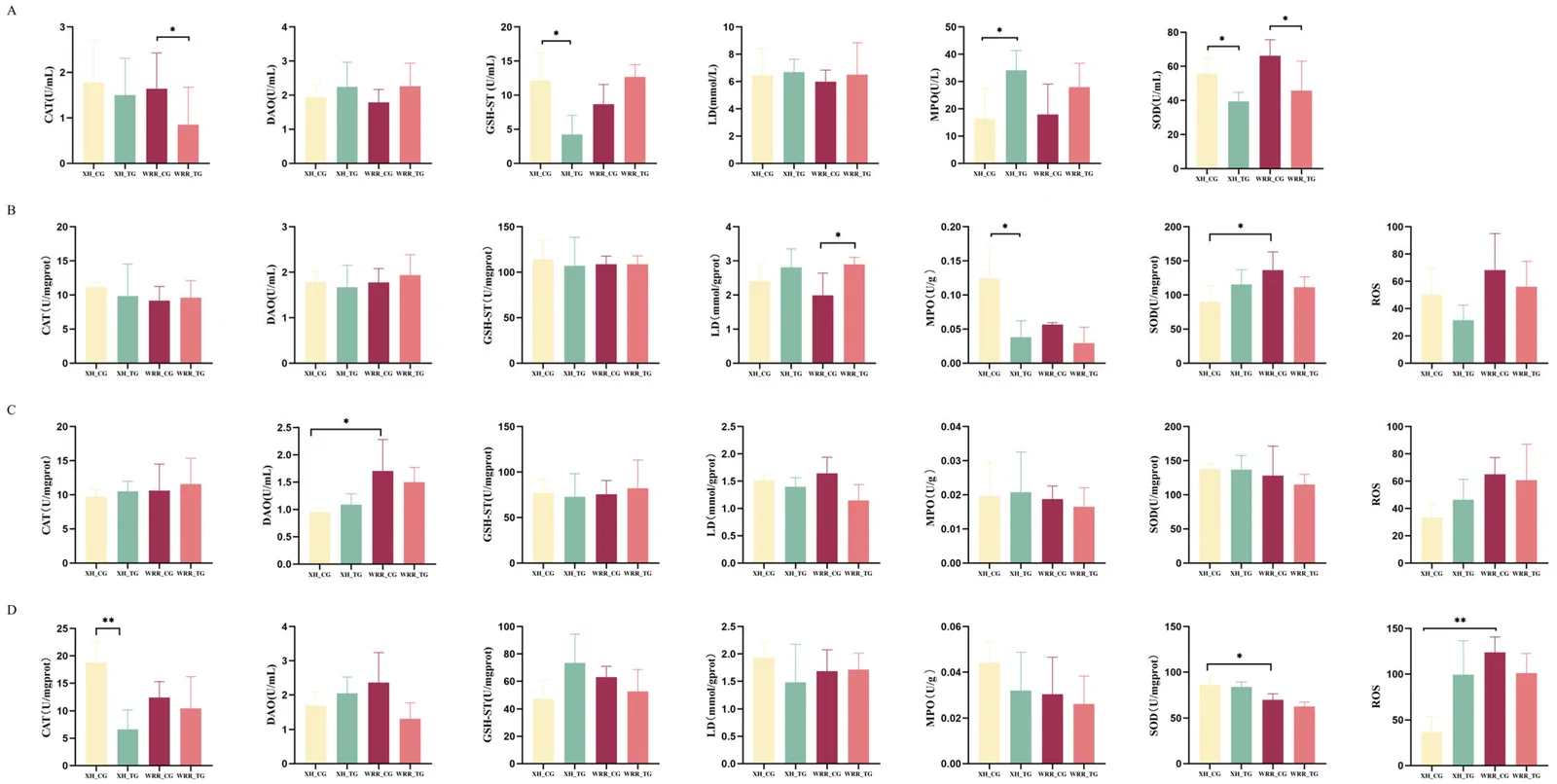
Impact of omnivorous feeding on antioxidant capacity in different tissues. Antioxidant indices in (**A**) serum, (**B**) duodenum, (**C**) jejunum, and (**D**) ileum are presented. Data are expressed as mean ± SEM (*n* = 6). Abbreviations: XH_CG, Xinghua chickens in the control group; XH_EG, Xinghua chickens in the omnivorous feeding group; WRR_CG, White Recessive Rock chickens in the control group; WRR_EG, White Recessive Rock chickens in the omnivorous feeding group. Asterisks indicate significant differences compared to the control group (* *p* < 0.05; ** *p* < 0.01).

As shown in Figure 1A, compared with the Xinghua control group, omnivorous feeding significantly decreased the activities of SOD (*p* = 0.038) and GSH-ST (*p* = 0.031) in serum while significantly increasing MPO activity (*p* = 0.029). Similarly, compared with the White Recessive Rock control group, omnivorous feeding significantly reduced the activities of SOD (*p* = 0.032) and CAT (*p* = 0.033) in serum. Except for GSH-ST, the other antioxidant parameters showed similar trends between the two breeds under omnivorous feeding.

In the duodenum, omnivorous feeding significantly decreased MPO activity in Xinghua chickens (*p* = 0.018) and significantly increased LD activity in White Recessive Rock chickens (*p* = 0.041), whereas the other indices showed no significant differences (Figure 1B).

In the jejunum, no significant differences were observed in any of the antioxidant indices between the omnivorous feeding and control groups (*p* = 0.043) (Figure 1C).

However, in the ileum, omnivorous feeding increased GSH-ST activity and decreased CAT activity in Xinghua chickens (*p* = 0.0052), whereas this feeding regime decreased DAO activity in White Recessive Rock chickens (Figure 1D).

### 3.2. Effects of Omnivorous Feeding and Breed Differences on Intestinal Morphology

**Figure 2:**
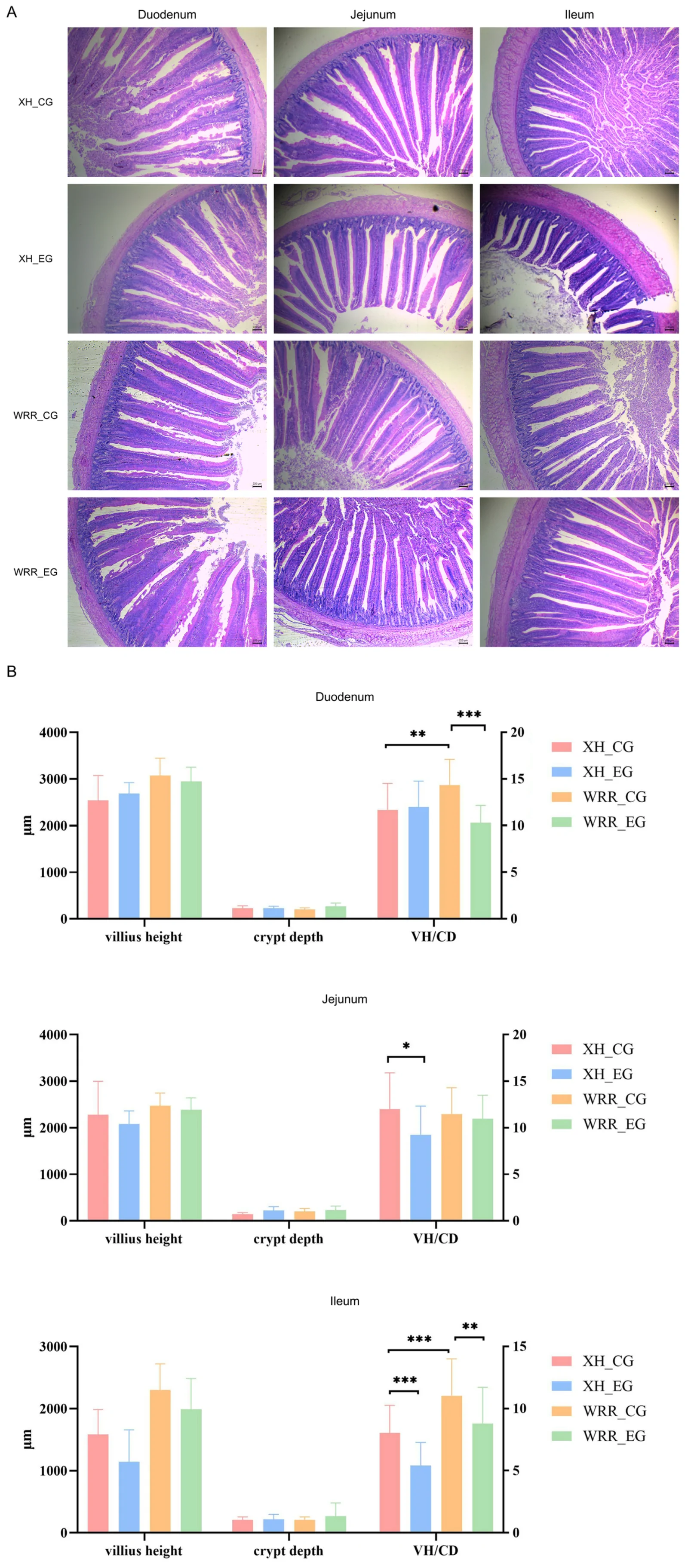
Effects of omnivorous feeding on intestinal morphological structure of 5-week-old broilers. (**A**) Paraffin sections of intestinal tissue stained with hematoxylin and eosin (H&E) at 5 weeks of age. Scale bar = 220 μm. (**B**) Quantification of villus height, crypt depth, and the villus height-to-crypt depth ratio (VH/CD). Data are expressed as mean ± SEM (*n* = 6). Abbreviations: XH_CG, Xinghua chickens in the control group; XH_EG, Xinghua chickens in the omnivorous feeding group; WRR_CG, White Recessive Rock chickens in the control group; WRR_EG, White Recessive Rock chickens in the omnivorous feeding group. Asterisks indicate significant differences compared to the control group (* *p* < 0.05; ** *p* < 0.01; *** *p* < 0.001).

**Figure 3:**
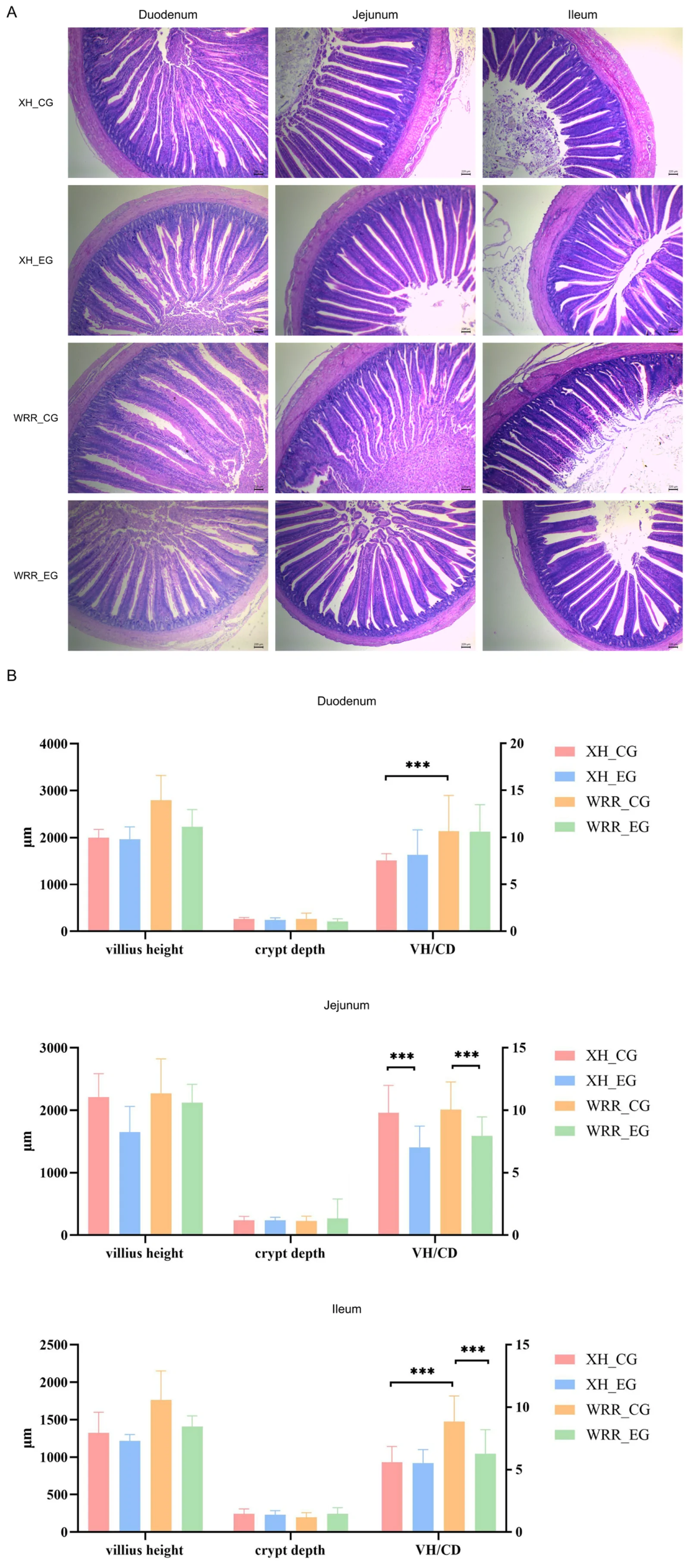
Effects of omnivorous feeding on intestinal morphological structure of 8-week-old broilers. (**A**) Paraffin sections of intestinal tissue stained with hematoxylin and eosin (H&E) at 8 weeks of age. Scale bar = 220 μm. (**B**) Quantification of villus height, crypt depth, and the villus height-to-crypt depth ratio (VH/CD). Data are expressed as mean ± SEM (*n* = 6). Abbreviations: XH_CG, Xinghua chickens in the control group; XH_EG, Xinghua chickens in the omnivorous feeding group; WRR_CG, White Recessive Rock chickens in the control group; WRR_EG, White Recessive Rock chickens in the omnivorous feeding group. Asterisks indicate significant differences compared to the control group (*** *p* < 0.001).

The histological sectioning results are shown in Figure 2 and Figure 3. By five weeks of age, the omnivorous feeding group of Xinghua chickens exhibited a significant reduction in the jejunal VH/CD ratio compared to the control group (*p* = 0.00028). Similarly, the omnivorous groups of White Recessive Rock chickens showed significant decreases in the VH/CD ratio in both the jejunum (*p* = 0.00035) and ileum (*p* = 0.00033). At eight weeks of age, the Xinghua chickens in the omnivorous group continued to exhibit a significant reduction in the jejunal VH/CD ratio compared to the control group (*p* = 0.023), whereas the omnivorous group of White Recessive Rock chickens showed a significant reduction in the VH/CD ratio in the duodenum (*p* = 0.00051) and ileum (*p* = 0.0072).

### 3.3. Effects of Omnivorous Feeding on the Gut Microbiota of Xinghua Chickens

At 5 weeks of age, alpha diversity analysis indicated that the ACE, Chao1, and Shannon indices in the duodenum were significantly different between the two groups. However, no significant differences were observed in other tissues. Beta diversity analysis showed clear separation of microbial communities in the duodenum, jejunum, ileum, and cecum. At the phylum level, omnivorous feeding did not alter the dominant bacteria, with *Firmicutes* remaining the predominant phylum (Figure S1). At the genus level, omnivorous feeding primarily reduced the relative abundance of *Lactobacillus* and *Bacillus* in the duodenum and jejunum, *Romboutsia* in the ileum, *Faecalibacterium* in the cecum, and *Enterococcus* and *Lactobacillus* in the feces (Figure S2).

At 8 weeks of age, significant differences were observed in the Simpson and Shannon indices of the cecal microbiota, indicating that omnivorous feeding specifically altered alpha diversity in the cecum. The two groups exhibited distinct clustering patterns, allowing for effective discrimination. *Firmicutes* remained the predominant phylum in both groups (Figure S3). Omnivorous feeding did not shift the dominant genera but primarily modulated their relative abundances. Specifically, the abundances of *Lactobacillus* and *Bacillus* were altered in the duodenum, jejunum, and ileum. Additionally, in the cecal tissue, the relative abundances of *Enterococcus* and *Shigella* were also affected (Figure S4). The results revealed specific taxa with significant abundance changes following omnivorous feeding: *Bacillus* and *Companilactobacillus* in the duodenum, *Bacillus* and *Romboutsia* in the jejunum, *g_Bacillus* and *Alistipes* in the cecum, and *Bacillus* and *Christensenellaceae_R_7_group* in the feces (Figure S5).

Omnivorous feeding significantly altered the relative abundances of *Lactobacillus*, *Bacillus*, unclassified *Bacteria*, unclassified *Muribaculaceae*, and *Romboutsia* in the gut microbiota of Xinghua chickens at 5 and 8 weeks (Figure S6). Pairwise comparisons of the jejunum microbiota between groups showed that omnivorous feeding led to a significant enrichment of pathways related to carbohydrate metabolism, amino acid metabolism, nucleotide metabolism, metabolism of cofactors and vitamins, and energy metabolism.

### 3.4. Effects of Omnivorous Feeding on the Gut Microbiota of White Recessive Rock Chickens

**Figure 4:**
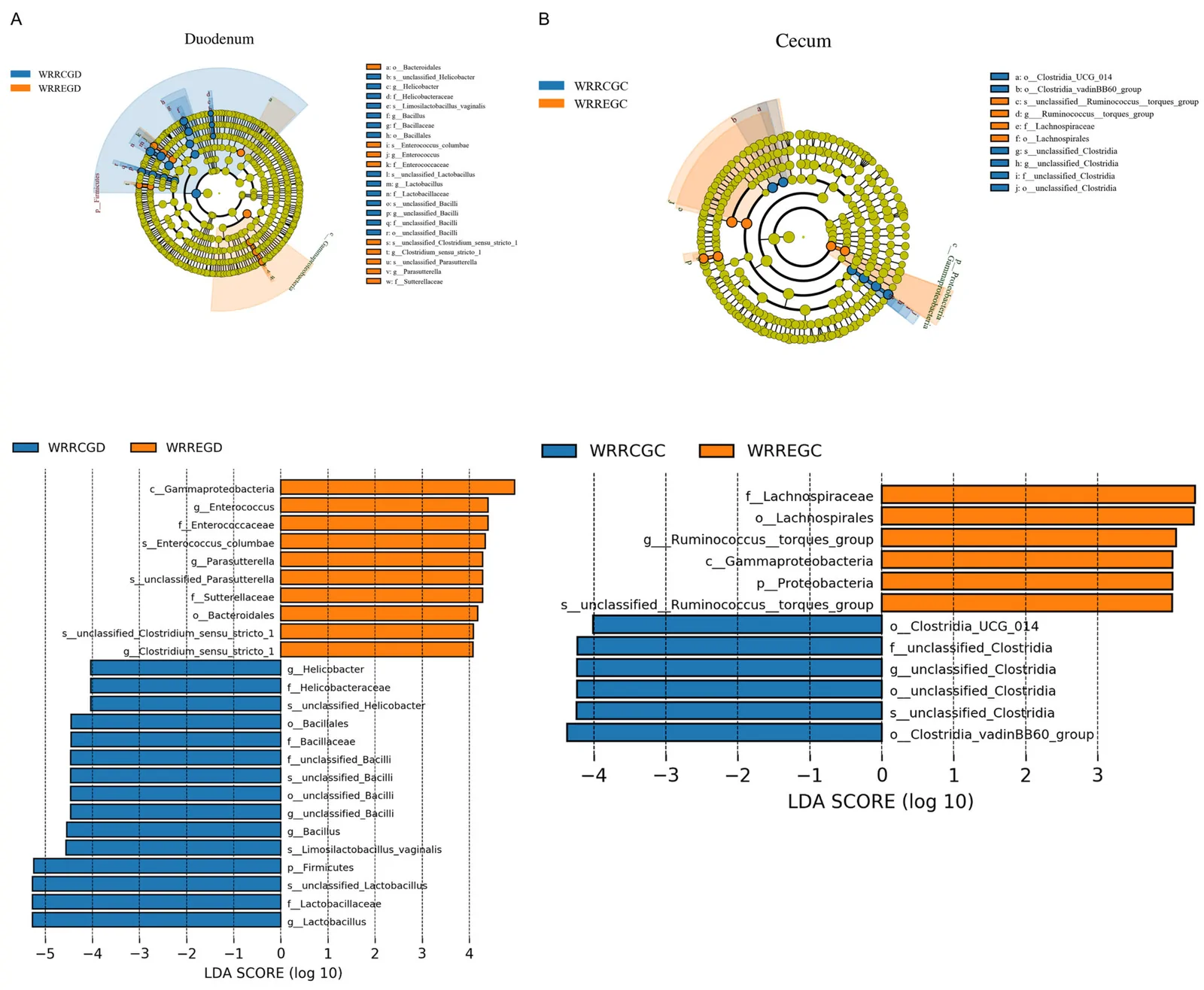
LEfSe analysis of differential gut microbiota in the duodenum and cecum of 5-week-old White Recessive Rock chickens. Linear discriminant analysis (LDA) scores indicate the magnitude of taxa associated with (**A**) duodenum and (**B**) cecum. Abbreviations: WRR_CG, White Recessive Rock chickens in the control group; WRR_EG, White Recessive Rock chickens in the omnivorous feeding group. Blue and red columns indicate control and experimental groups, respectively.

At 5 weeks of age, comparative analysis between the two groups of White Recessive Rock chickens revealed no significant differences in species richness or alpha diversity across the various tissues. Principal Coordinates Analysis (PCoA) showed that the microbial communities in the jejunum, ileum, and feces clustered closely together. In contrast, the duodenum and cecum samples from the two groups formed separate clusters, indicating compositional differences in these segments. At the phylum level, omnivorous feeding did not alter the dominant bacterial phyla, with *Firmicutes* remaining the predominant phylum (Figure S7). At the genus level, the dominant genera in the duodenum and jejunum remained *Lactobacillus* in both groups. However, in the ileum, the dominant genus shifted from *Lactobacillus* to *Candidatus Arthromitus* due to omnivorous feeding. In the cecum, the dominant genus shifted from *Lactobacillus* to *Escherichia-Shigella*. In the fecal microbiota, the dominant genus shifted from *Faecalibacterium* to *Bacteroides*. Furthermore, omnivorous feeding primarily reduced the relative abundance of *Lactobacillus* and *Bacillus* in the duodenum, jejunum, ileum, and cecum, as well as decreasing the abundance of *Faecalibacterium* in the feces (Figure S8). Significant differences in the duodenum were primarily observed in the genera *Enterococcus*, *Parasutterella*, *Clostridium sensu stricto 1*, *Helicobacter*, unclassified *Bacilli*, *Bacillus*, and *Lactobacillus*. In the cecum, significant differences were mainly found in *Ruminococcus torques group* and unclassified *Clostridia* (Figure 4).

**Figure 5:**
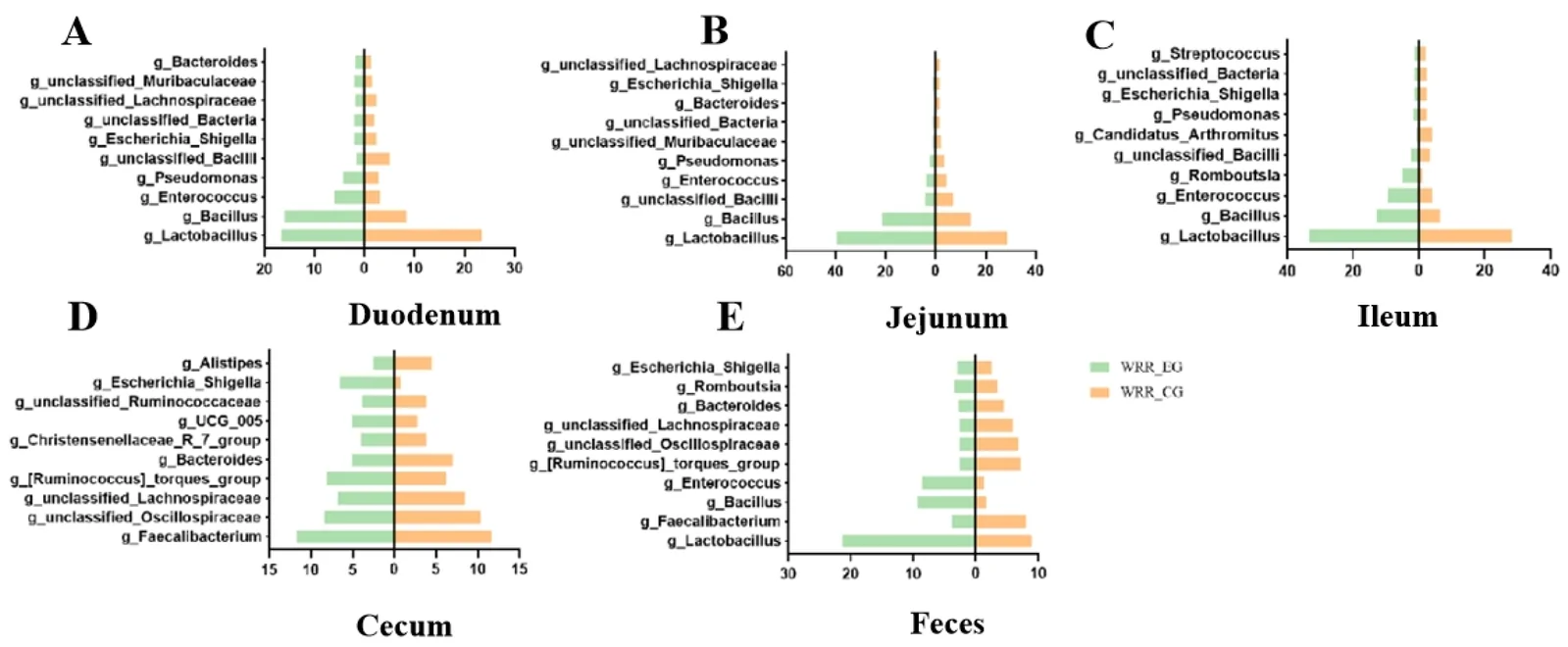
Effects of omnivorous feeding on the gut microbiota composition of 8-week-old White Recessive Rock chickens at the genus level. Relative abundance of differential genera in the (**A**) duodenum, (**B**) jejunum, (**C**) ileum, (**D**) cecum, and (**E**) feces. Abbreviations: WRR_CG, White Recessive Rock chickens in the control group; WRR_EG, White Recessive Rock chickens in the omnivorous feeding group.

**Figure 6:**
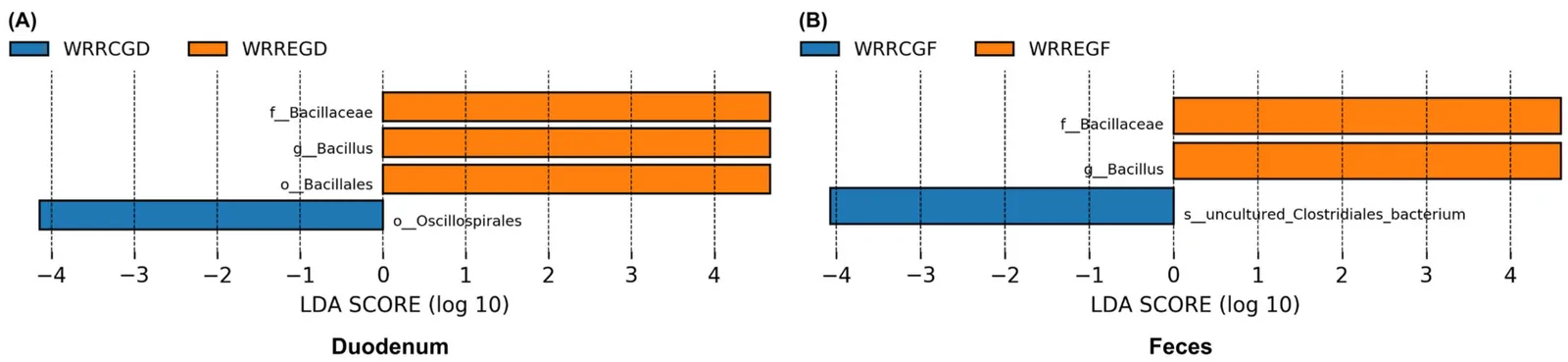
LEfSe analysis of differential gut microbiota in the duodenum and feces of 8-week-old White Recessive Rock chickens. Linear discriminant analysis (LDA) scores of differential taxa in the (**A**) duodenum and (**B**) feces. Abbreviations: WRR_CG, White Recessive Rock chickens in the control group; WRR_EG, White Recessive Rock chickens in the omnivorous feeding group.

At 8 weeks of age, comparative analysis between the omnivorous and control groups of White Recessive Rock chickens revealed that alpha diversity metrics showed no significant differences across various tissues. PCoA demonstrated distinct clustering of microbial communities between the two groups in most intestinal segments, except for the duodenum and ileum, suggesting that omnivorous feeding induced significant structural differentiation in specific gut regions. At the phylum level, Firmicutes remained the predominant taxa in both groups, and omnivorous feeding did not change this dominant status (Figure S9). At the genus level, the abundances of *Lactobacillus* and *Bacillus* were altered in the duodenum, jejunum, and ileum. The abundance of *Escherichia-Shigella* increased in the cecum. In the fecal microbiota, the abundances of *Lactobacillus*, *Bacillus*, and *Enterococcus* were elevated, while the abundance of *Faecalibacterium* decreased (Figure 5). LEfSe analysis indicated significant changes in *Bacillus* abundance in the duodenum and feces (Figure 6).

**Figure 7:**
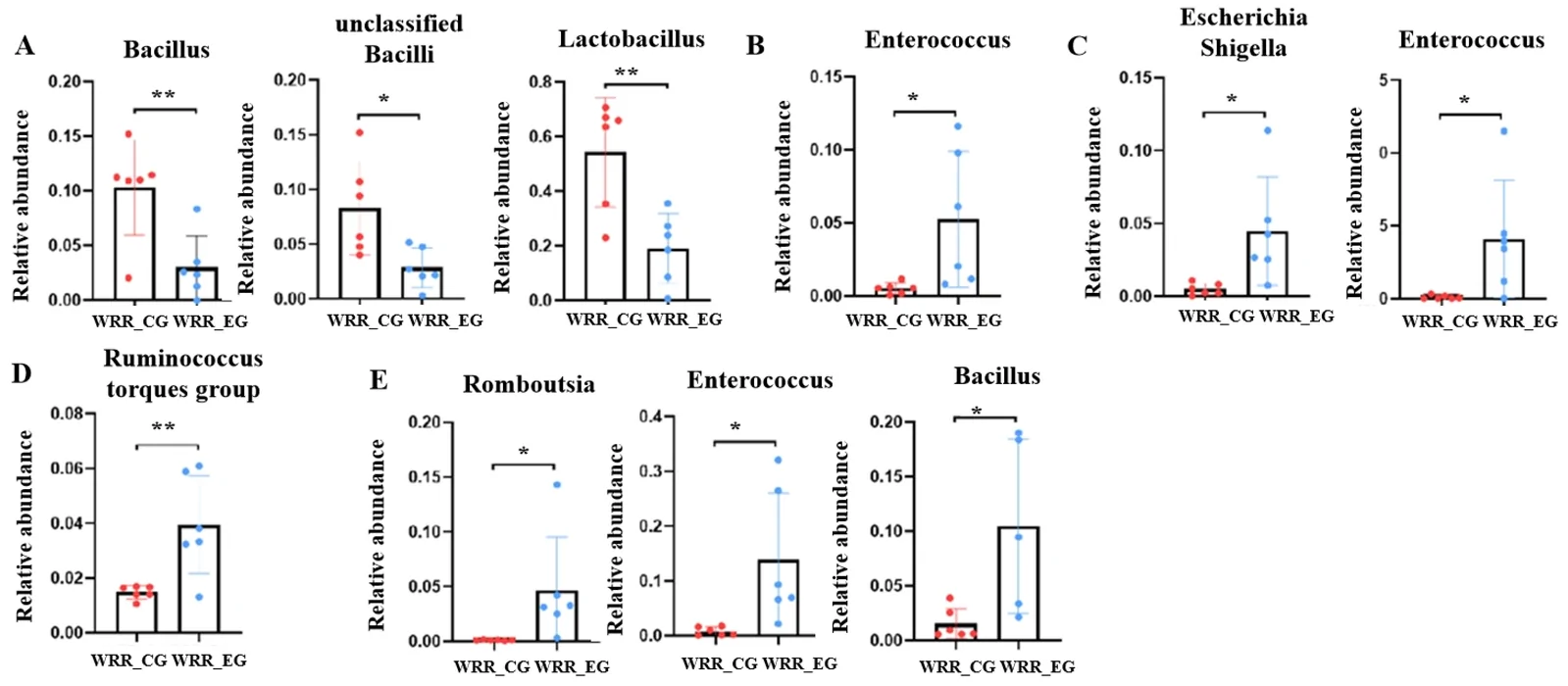
Comparison of the relative abundance of differential gut microbiota between the two groups of White Recessive Rock chickens at the genus level. The relative abundance of differential genera in the (**A**) duodenum, (**B**) jejunum, (**C**) ileum, (**D**) cecum, and (**E**) feces is shown. Data are presented as mean ± SEM. * *p* < 0.05, ** *p* < 0.01.

Integrative analysis of data from 5 and 8 weeks of age revealed that omnivorous feeding significantly modulated the relative abundances of key bacterial genera in the gut of White Recessive Rock chickens, including *Bacillus* (*p* = 0.0047 in the duodenum; *p* = 0.037 in the feces), *Enterococcus* (*p* = 0.037 in the jejunum; *p* = 0.028 in the feces), *Escherichia-Shigella* (*p* = 0.017), and *Romboutsia* (*p* = 0.0015) (Figure 7). Functional predictions based on the KEGG database indicated that omnivorous feeding led to a significant enrichment of metabolic pathways associated with carbohydrate, amino acid, and nucleotide metabolism in the jejunal microbiota.

### 3.5. Association Analysis of the Microbiome and Meat Quality

To investigate the correlation between the gut microbiome and muscle quality traits, we performed a Spearman correlation analysis. We analyzed the association between meat quality indicators previously measured and the top 20 most abundant bacterial taxa in the duodenum, jejunum, ileum, cecum, and feces of 8-week-old broilers.

**Figure 8:**
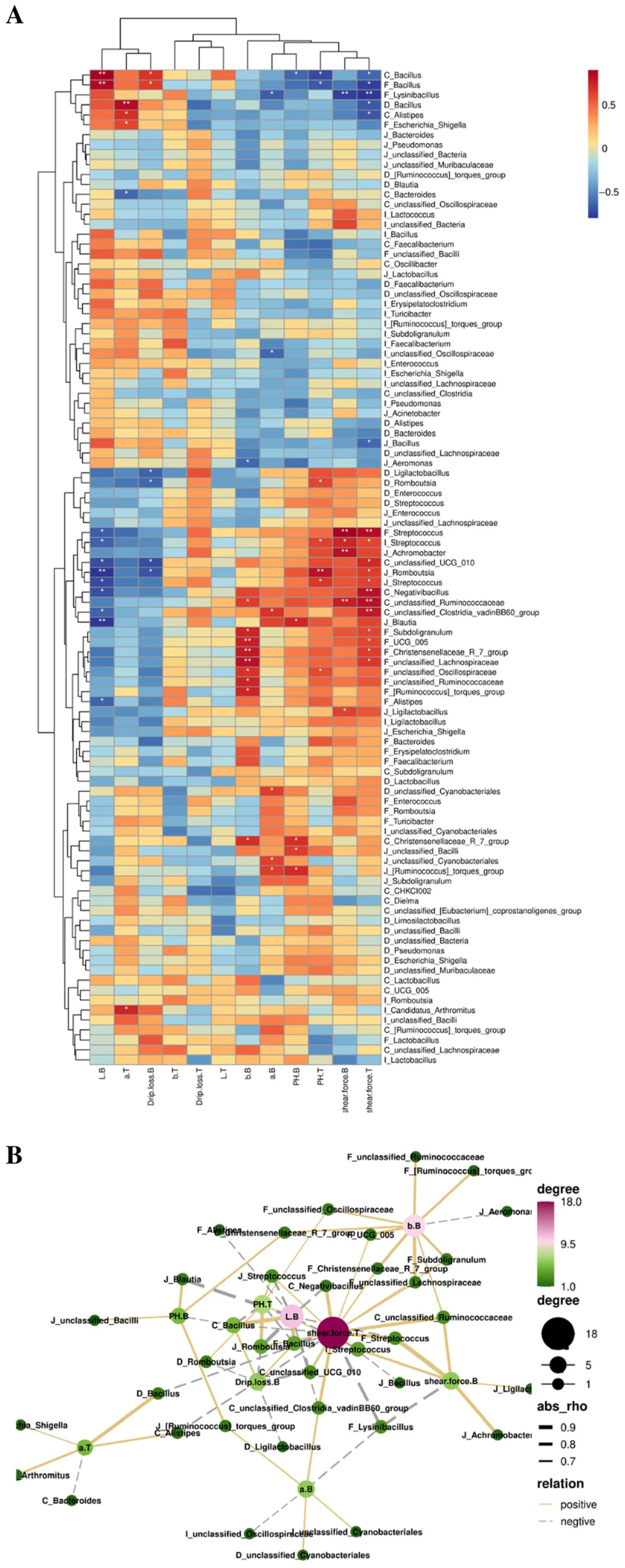
Correlation analysis between gut microbiota and meat quality traits in Xinghua chickens. The relationships between differential bacterial genera and meat quality parameters were evaluated using the Spearman rank correlation coefficient. To ensure statistical rigor and control the false discovery rate (FDR) arising from multiple hypothesis testing, raw *p*-values were adjusted using the Benjamini–Hochberg (BH) procedure. (**A**) Heatmap displaying the Spearman correlation coefficients. The color intensity represents the magnitude of the correlation, ranging from negative (blue) to positive (red). Only correlations with an adjusted *p*-value (*p* < 0.05) are displayed. (**B**) Network diagram illustrating significant interactions between key microbiota and meat quality parameters. Nodes represent bacterial genera (circles) and meat quality traits (rectangles). Edges indicate significant correlations (*p* < 0.05), with line thickness proportional to the correlation coefficient (|r|). * *p* < 0.05, ** *p* < 0.01.

In Xinghua chickens, *Bacillus* was found to be closely associated with chicken meat quality. It showed a significant positive correlation with breast muscle lightness (*L*.*B*), pH, and drip loss in the duodenum, jejunum, cecum, and feces while exhibiting a significant negative correlation with pH and leg muscle shear force. Specific correlations were observed in different intestinal segments. In the duodenum, *Bacillus*, *Ligilactobacillus*, and *Romboutsia* were the primary genera significantly affecting meat quality. In the jejunum, *Bacillus*, *Aeromonas*, *Achromobacter*, *Romboutsia*, *Streptococcus*, *Blautia*, and *Ligilactobacillus* showed significant associations. Notably, *Romboutsia* was significantly negatively correlated with breast muscle lightness and drip loss but positively correlated with leg muscle pH and shear force. In the ileum, Unclassified *Oscillospiraceae*, *Streptococcus*, and *Candidatus Arthromitus* showed significant correlations. In the cecum, significant correlations were observed with *Bacillus*, *Alistipes*, *Bacteroides*, Unclassified *UCG 010*, *Negativibacillus*, unclassified *Ruminococcaceae*, and unclassified *Clostridia vadinBB60*. In the feces, a larger number of microbiota were associated with meat quality, such as *Bacillus*, *Lysinibacillus*, *Escherichia*-*Shigella*, *Streptococcus*, *Subdoligranulum*, *UCG 005*, *Christensenellaceae R7*, Unclassified *Lachnospiraceae*, Unclassified *Oscillospiraceae*, Unclassified *Ruminococcaceae*, [*Ruminococcus*] *torques group*, and *Alistipes*. Most of these fecal microbiota were positively correlated with breast muscle yellowness (*b*.*B*) and leg muscle shear force (Figure 8).

**Figure 9:**
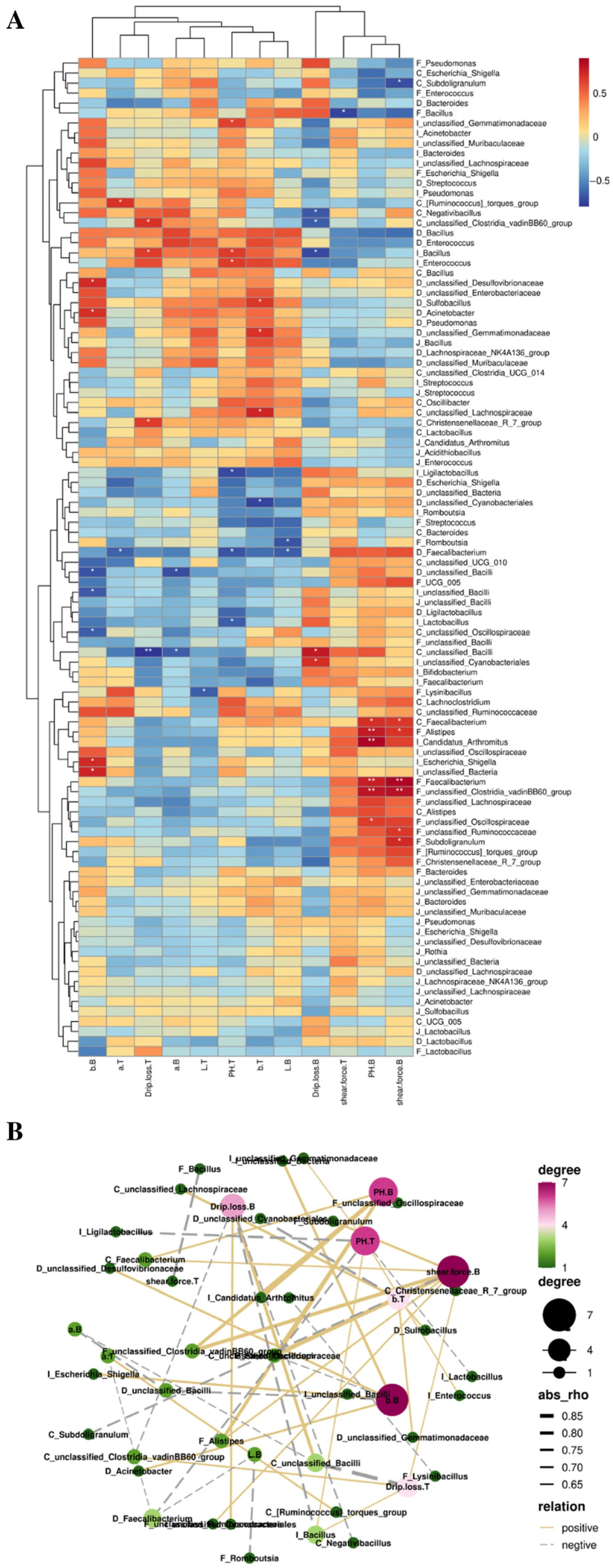
Correlation analysis between gut microbiota and meat quality traits in White Recessive Rock chickens. The relationships between differential bacterial genera and meat quality parameters were evaluated using the Spearman rank correlation coefficient. To ensure statistical rigor and control the false discovery rate (FDR) arising from multiple hypothesis testing, raw *p*-values were adjusted using the Benjamini–Hochberg (BH) procedure. (**A**) Heatmap displaying the Spearman correlation coefficients. The color intensity represents the magnitude of the correlation, ranging from negative (blue) to positive (red). Only correlations with an adjusted *p*-value (*p* < 0.05) are displayed. (**B**) Network diagram illustrating significant interactions between key microbiota and meat quality parameters. Nodes represent bacterial genera (circles) and meat quality traits (rectangles). Edges indicate significant correlations (*p* < 0.05), with line thickness proportional to the correlation coefficient (|r|). * *p* < 0.05, ** *p* < 0.01.

In the White Recessive Rock chickens, no bacterial genera among the top 20 most abundant in the jejunum were found to be significantly associated with meat quality traits. *Faecalibacterium* showed significant correlations with meat quality in the duodenum, cecum, and feces; in the cecum and feces, it was positively correlated with breast muscle pH and shear force. In the duodenum, it was significantly negatively correlated with leg muscle yellowness (*b.B*) and pH. Additionally, several other genera showed significant associations related to meat quality, such as unclassified *Desulfovibrionaceae*, *Sulfobacillus*, *Acinetobacter*, unclassified *Gemmatimonadaceae*, and unclassified *Cyanobacteriales* in the duodenum; unclassified *Gemmatimonadaceae*, *Bacillus*, *Enterococcus*, *Ligilactobacillus*, *Candidatus Arthromitus*, and *Lactobacillus* in the ileum; *Subdoligranulum*, [*Ruminococcus*] *torques*, *Negativibacillus*, unclassified *Clostridia vadinBB60*, *Christensenellaceae R 7*, Unclassified *Lachnospiraceae*, and unclassified *Bacilli* in the cecum; and *Bacillus*, *Romboutsia*, *Lysinibacillus*, and *Alistipes* in the feces (Figure 9).

## 4. Discussion

### 4.1. Effects of Omnivorous Feeding on Antioxidant Capacity in Serum and Intestinal Tissues of Xinghua and White Recessive Rock Chickens

In this experiment, omnivorous feeding significantly altered antioxidant indices in both serum and intestinal tissues. Omnivorous feeding significantly decreased the levels of SOD and GSH-ST while significantly increasing MPO levels in the serum of Xinghua chickens. Concurrently, SOD and CAT levels in the serum of White Recessive Rock chickens also showed a significant decline. These findings indicate that this feeding strategy might enhance lipid peroxidation and compromise systemic antioxidant defenses. A decrease in MPO content was observed only in the duodenum of Xinghua chickens, suggesting an alleviated intestinal inflammatory response. In contrast, LD levels increased significantly in White Recessive Rock chickens. No significant differences were observed in any indices across the jejunum groups, indicating that omnivorous feeding has minimal impact on the antioxidant capacity of the jejunum. An increase in GSH-ST coupled with a decrease in CAT was observed in the ileum of Xinghua Chickens, a result consistent with that of previous studies [17,18], potentially protecting tissue cells from oxidative damage. A significant decrease in DAO content was found in the ileum of omnivorous White Recessive Rock chickens. Since impaired intestinal mucosal barrier function typically leads to a massive release of DAO into the bloodstream or intestinal lumen [19], the reduced DAO levels suggest that omnivorous feeding did not impair intestinal barrier function in these broilers. Comparative analysis revealed that SOD activity in the duodenum, DAO activity in the jejunum, and ROS levels in the ileum of Xinghua chickens were significantly lower than those in White Recessive Rock chickens. This suggests that White Recessive Rock chickens might be more susceptible to oxidative stress than Xinghua chickens under omnivorous feeding conditions. However, further experimental data are needed to verify this.

### 4.2. Effects of Breed Differences and Omnivorous Feeding on Intestinal Morphological Structure

At 5 and 8 weeks of age, the VH/CD ratios in the duodenum and ileum of White Recessive Rock chickens were significantly higher than those of Xinghua chickens. These results suggest that fast-growing broilers possessed a higher VH/CD ratio, which helped increase the effective absorption area of the intestinal mucosa, thereby accelerating the uptake and utilization of nutrients. The VH/CD ratios in the jejunum of omnivorous-fed Xinghua chickens and in the jejunum and ileum of omnivorous-fed White Recessive Rock chickens were significantly lower than those in their respective control groups. Consistent with the findings of this study, dietary supplementation has been shown to significantly increase the VH/CD ratio across various intestinal segments, reduce crypt depth, and thereby enhance the absorptive capacity of the small intestine in animals [20,21]. Since the duodenum, jejunum, and ileum serve as the primary sites for food digestion and nutrient absorption in livestock and poultry, the histomorphological characteristics of the small intestinal mucosa form the structural basis of their physiological functions [22]. Specifically, VH, CD, and VH/CD are potential indicators for assessing intestinal digestive and absorptive capacity, reflecting the efficiency of nutrient utilization. Generally, a higher VH/CD ratio might indicate a larger mucosal absorption area and a stronger capacity for nutrient absorption. This suggests that the specific impact of omnivorous feeding on intestinal morphology might depend on the type and proportion of unconventional feed ingredients used.

In this study, the observed reduction in the villus height-to-crypt depth ratio (V/C) under omnivorous feeding conditions might stem from histological remodeling of the intestine in response to dietary changes. Omnivorous feeding introduced a higher proportion of cellulose and unconventional agricultural by-products, which not only increased dietary complexity but might also cause mild irritation to the intestinal mucosa due to potential anti-nutritional factors and the coarse physical structure. This persistent physical stimulation or digestive burden might accelerate crypt cell proliferation and increase crypt depth, leading to a decreased V/C ratio. This study revealed that White Recessive Rock chickens (fast-growing broilers) exhibit a significantly higher sensitivity to dietary changes than Xinghua chickens (local breeds). This was specifically manifested in a broader range of affected intestinal segments, involving both the jejunum and ileum, resulting in a more pronounced decrease in the villus height-to-crypt depth ratio (V/C). The underlying reason for this might be that, having undergone intensive breeding, the White Recessive Rock chickens were easily disrupted by omnivorous feeding, as they usually maintain an intestinal state of high-efficiency absorption and a physiological balance. In contrast, Xinghua chickens demonstrated a stronger capability to maintain intestinal homeostasis.

This indicates that it is essential to strictly maintain the stability of the dietary formulation while raising fast-growing broilers such as White Recessive Rock chickens. In contrast, for local breeds like the Xinghua chicken, more flexible feeding strategies could be adopted. Specifically, their tolerance to omnivorous feeding could be exploited to reduce feeding costs or improve meat quality. Furthermore, these findings suggest that local chicken breeds, such as the Xinghua chicken, might harbor superior genetic determinants or microbial resources for regulating intestinal health. These resources are worthy of in-depth exploration and could be harnessed for the genetic improvement of modern poultry breeding programs.

### 4.3. Effects of Breed Differences and Omnivorous Feeding on Intestinal Microbiota

Based on 16S rRNA sequencing, this study systematically analyzed the impact of omnivorous feeding on the gut microbiota in various intestinal segments (the duodenum, jejunum, ileum, and cecum) and feces of Xinghua chickens (local breed) and White Recessive Rock chickens. The results revealed significant breed-specific responses to omnivorous feeding. In Xinghua chickens, the diversity and richness of the microbiota in the duodenum and cecum were significantly altered. In contrast, in White Recessive Rock chickens, changes were limited to shifts in the relative abundance of specific taxa, while the overall microbial diversity and richness remained relatively stable. The gut microbiota constitutes a highly plastic and complex ecosystem, whose structure and function are dually modulated by the host’s genetic background and environmental factors, particularly dietary composition [23]. Alterations in this microbial community could influence the expression of amino acid and ion transporters, thereby modulating the growth and development of broilers [24].

In this study, the predominant phyla in the gut of both chicken breeds were identified as *Firmicutes*, *Proteobacteria*, and *Bacteroidota*, which is consistent with previous findings. Functionally, members of the *Firmicutes* can influence carbohydrate metabolism and degrade insoluble polysaccharides (e.g., cellulose) into soluble oligosaccharides. In contrast, *Bacteroidota* are involved in various essential metabolic activities, including carbohydrate fermentation, utilization of nitrogenous substances, and biotransformation of bile acids and other steroids [25].

Omnivorous feeding was observed to significantly increase the relative abundance of *Lactobacillus* and *Bacillus* in both Xinghua and White Recessive Rock chickens at 8 weeks of age. As predominant probiotics in the chicken gut microbiota, these genera can synthesize various beneficial metabolites, including vitamins (e.g., vitamin K and B-complex vitamins), short-chain fatty acids (e.g., acetate, butyrate, and propionate), organic acids (e.g., lactic acid), and antimicrobial compounds (e.g., bacteriocins). These metabolites contribute to lowering host triglyceride levels and inducing non-pathogenic immune responses [26], thereby playing a crucial role in maintaining microbial homeostasis and suppressing potential pathogens [5].

Notably, the cecum, which harbors the most abundant microbial community in poultry [27], exhibited a distinct shift in response to omnivorous feeding, which was characterized by an increased abundance of *Escherichia-Shigella*, *UCG-005*, and *Alistipes*. It has been reported that UCG-005, *Alistipes*, and *Clostridium sensu stricto 1* could accelerate lipid deposition, thereby increasing backfat thickness in Enshi Black pigs [28].

Pairwise comparison analysis revealed distinct differentially abundant genera in response to omnivorous feeding between the two chicken breeds. In Xinghua chickens, the key differential genera primarily included *Bacillus*, *Romboutsia*, *Alistipes*, *Christensenellaceae R7*, *Lactiplantibacillus, Turicibacter*, *Delftia*, unclassified *Clostridia vadinBB60*, and *Cellvibrio*. In contrast, the key differential genera in White Recessive Rock chickens mainly included *Enterococcus*, *Parasutterella*, *Clostridium sensu stricto1*, *Helicobacter*, *Bacillus*, *Lactobacillus*, and *Ruminococcus torques*.

In particular, the enrichment of Romboutsia induced by omnivorous feeding has significant physiological implications. Previous studies demonstrated that *Romboutsia* was correlated with intestinal permeability and fat deposition [29], suggesting its potential role in meat quality. Furthermore, the modulation of *Christensenellaceae R7* is of particular interest, as this taxon has been reported to be negatively associated with fat deposition, colitis, and metabolic syndromes [30]. Additionally, the taxonomic groups associated with the aforementioned microbiota could induce the production of the anti-inflammatory cytokine IL-10 in peripheral blood mononuclear cells, enhance tight junction protein expression, and reduce intestinal mucosal permeability, thereby alleviating gut inflammation [31].

### 4.4. The Relationship Between Gut Microbiota and Meat Quality

Our study on the correlation between the gut microbiota and meat quality revealed that taxa in the jejunum, such as *Ligilactobacillus*, *Bacteroides*, *Pseudomonas*, *Acinetobacter*, unclassified *Bacteria*, and *Escherichia-Shigella*, showed significant negative correlations with leg muscle lightness, yellowness, pH, and shear force. Similarly, the ileal microbiota, such as *Bacillus*, *Faecalibacterium*, *Bacteroides*, *Pseudomonas*, unclassified *Gemmatimonadaceae*, *Acinetobacter*, *Escherichia-Shigella*, unclassified *Bacteria*, and *Lactobacillus*, were significantly negatively correlated with pH, shear force, lightness, yellowness, and drip loss.

Furthermore, significant associations were identified with unclassified *Lachnospiraceae* and *Alistipes* in the duodenum; *Oscillibacter*, unclassified *Ruminococcaceae*, and *Negativibacillus* in the cecum; and *Streptococcus*, *Enterococcus*, *Lysinibacillus*, and *Escherichia-Shigella* in the feces. These bacteria might represent potential key microbiota underlying the divergence in meat quality and flavor between local and white-feathered broiler chickens.

In brief, these findings indicate that shifts in the microbial community could profoundly influence growth performance, immune function, and meat quality in poultry [32]. However, further investigations are warranted to elucidate the specific mechanisms by which such microbial alterations affect nutrient absorption in the host.

## 5. Conclusions

In conclusion, this study demonstrates that omnivorous feeding could significantly modulate antioxidant capacity, intestinal morphology, and gut microbiota composition in both White Recessive Rock and Xinghua chickens. This feeding regimen changed the VH/CD ratio and simultaneously altered beneficial microbial taxa such as *Bacillus* and *Romboutsia*, thereby regulating metabolic functions related to carbohydrate, amino acid, and energy metabolism. Crucially, specific genera, including *Faecalibacterium*, *Bacillus*, and *Romboutsia*, were identified as key factors associated with meat quality traits. These findings suggest that strategic omnivorous feeding offers a promising approach to optimizing intestinal health and improving meat quality through microbiota-mediated mechanisms.

## Data Availability

The original data presented in the study are included in the article. Further inquiries can be made to the corresponding author.

## Supplementary Materials

The following supporting information can be downloaded at: https://www.mdpi.com/article/10.3390/ani16213392/s1. Table S1: Chemical composition of experimental diets (on an as-fed basis); Table S2: 16S amplified fragment; Table S3: Reaction mixture for V3-V4 region; Table S4: 1P Reaction Program; Table S5: 2P Reaction System for V3-V4 Region; Table S6: 2P Reaction Program; Figure S1: Effects of mixed feeding on the differential gut microbiota of 5-week-old Xinghua chickens at the phylum level; Figure S2: Effects of mixed feeding on the differential gut microbiota of 5-week-old Xinghua chickens at the genus level; Figure S3: Effects of mixed feeding on the differential gut microbiota of 8-week-old Xinghua chickens at the phylum level; Figure S4: Effects of mixed feeding on the differential gut microbiota of 8-week-old Xinghua chickens at the genus level; Figure S5: LDA scores of differential gut microbiota in 8-week-old Xinghua chickens; Figure S6: Partial differential genera between the two groups of Xinghua chickens; Figure S7: Effects of mixed feeding on the differential gut microbiota of 5-week-old White Recessive Rock chickens at the phylum level; Figure S8: Effects of mixed feeding on the differential gut microbiota of 5-week-old White Recessive Rock chickens at the genus level; Figure S9: Effects of mixed feeding on the differential gut microbiota of 8-week-old White Recessive Rock chickens at the phylum level.
