# Supplementary material for: The Impact of Omnivorous Feeding on Antioxidant Capacity, Gut Microbiota, and the Relationship Between Gut Microbiota and Meat Quality in Xinghua and White Recessive Rock Chickens
Source: Animals (Basel). 2026 Oct 27;16(21):3392. doi: 10.3390/ani16213392 (PMC13761735; doi:10.3390/ani16213392)
Supplement: Supplementary file 1 [file animals-16-03392-s001.zip › animals-4575534-supplementary.pdf]

Table S1. Chemical composition of experimental diets (on an as-fed basis)

Component	Standard Diet (Chia Tai)	Omnivorous Diet
**Ingredient (%)**		
Corn	Proprietary	-
Soybean meal	Proprietary	-
... [Other ingredients]	Proprietary	-
Rice	-	50
Rice husks	-	30
Fresh vegetables	-	20
**Nutrient content (analyzed)**		
Crude protein (%)	21.0	18.67
Crude fiber (%)	6.00	11.67
Total ash (%)	8.0	5.83
Calcium (%)	0.6-1.20	0.92
Phosphorus (%)	0.5	0.73
Sodium chloride (%)	0.2-0.8	0.43

Table S2. 16S amplified fragment

Amplified Region	Primer Name	Primer Sequence (5’-3’)
V3-V4	338F	ACTCCTACGGGAGGCAGCA
	806R	GGACTACHVGGGTWTCTAAT

Table S3. Reaction mixture for V3-V4 region

Reagent	Volume（μL）
DNA	1
Indicator Internal Control	1
NFW	0.2
KOD FX	0.2
KOD buffer	5
2 mM dNTP	2
Barcode Primer（10 μM）	0.6
Total Volume	10

Table S4. 1P Reaction Program

Temperature	Time	Cycle
95℃	5 min	
95℃	30 sec	
50℃	30 sec	20 cycles
72℃	40 sec	
72℃	7 min	
4℃	∞	

Table S5. 2P Reaction System for V3-V4 Region

Reagent	Volume（μL）
Purified Product of 1st PCR	5
MPPI-a	2.5
MPPI-b	2.5
2×Q5 HF MM	10
Total	20

Table S6. 2P Reaction Program

Temperature	Time	Cycle
98℃	30 sec	
98℃	10 sec	
65℃	30 sec	10 cycles
72℃	30 sec	
72℃	5 min	
4℃	∞	

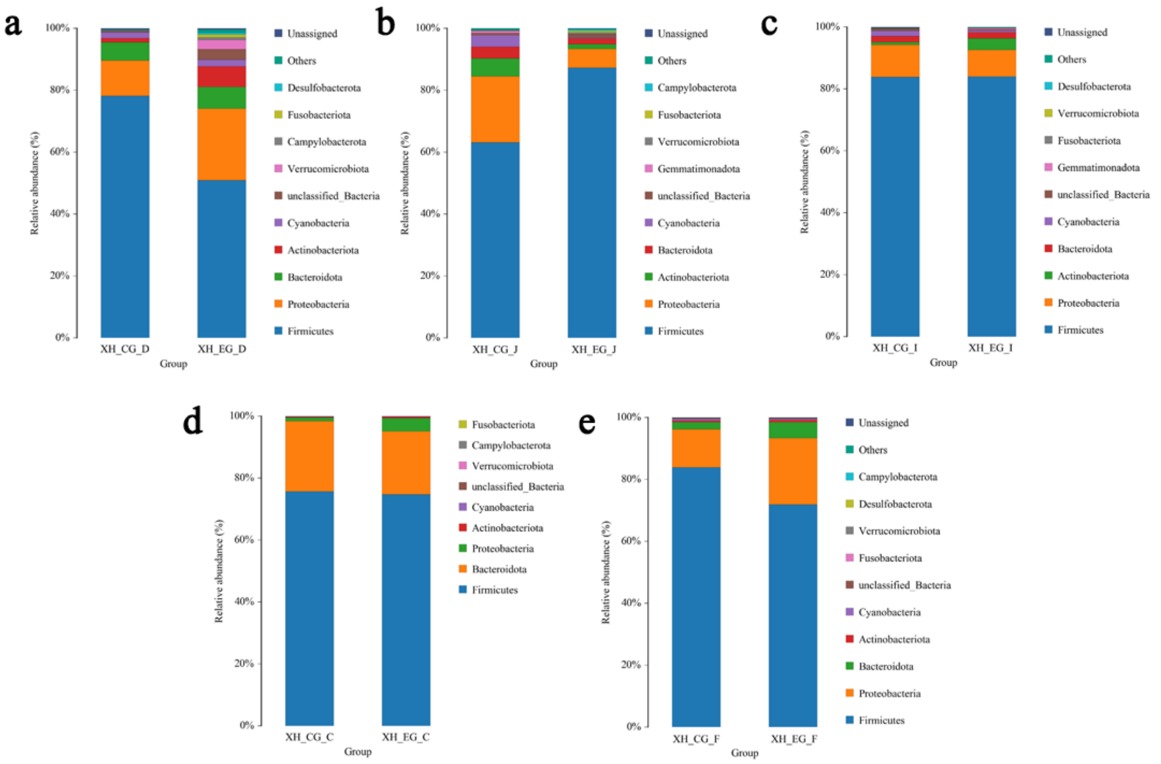

Figure S1. Effects of mixed feeding on the differential gut microbiota of 5-week-old Xinghua chickens at the phylum level. The microbial differences at the phylum level was shown in the duodenum (A), jejunum (B), ileum (C), cecum (D), and feces (E), respectively. XH_CG represents the control group of Xinghua chickens, and XH_EG represents the experimental group of Xinghua chickens.

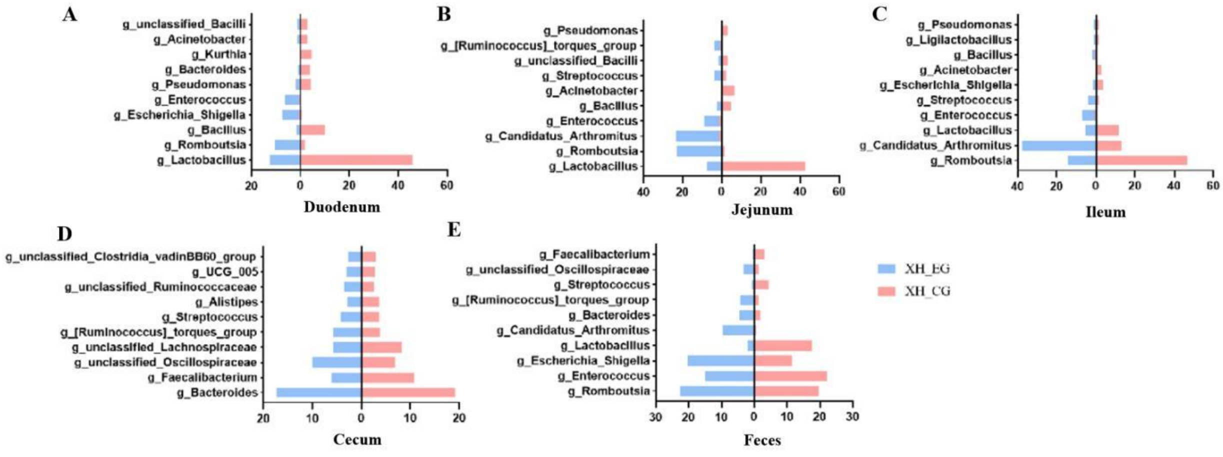

Figure S2. Effects of mixed feeding on the differential gut microbiota of 5-week-old Xinghua chickens at the genus level. The microbial differences at the genus level was shown in the duodenum (A), jejunum (B), ileum (C), cecum (D), and

feces (E), respectively. XH_CG represents the control group of Xinghua chickens, and XH_EG represents the experimental group of Xinghua chickens.

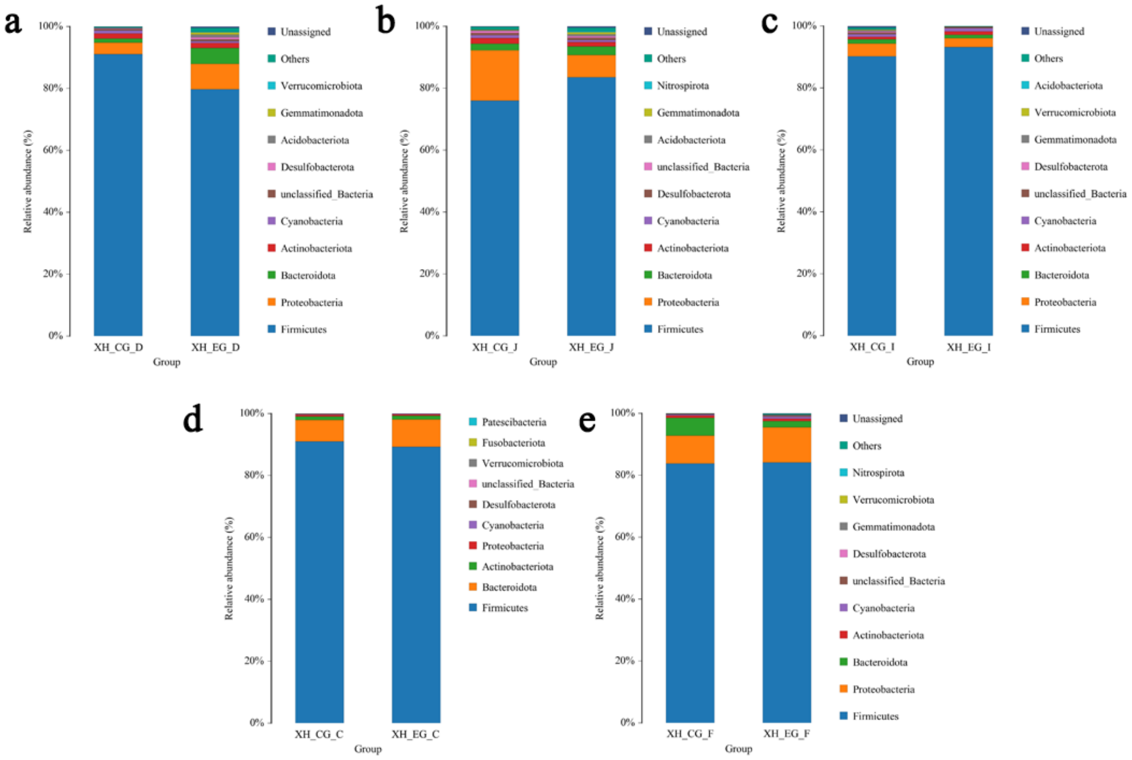

Figure S3. Effects of mixed feeding on the differential gut microbiota of 8-week-old Xinghua chickens at the phylum level. The microbial differences at the phylum level was shown in the duodenum (A), jejunum (B), ileum (C), cecum (D), and feces (E), respectively. XH_CG represents the control group of Xinghua chickens, and XH_EG represents the experimental group of Xinghua chickens.

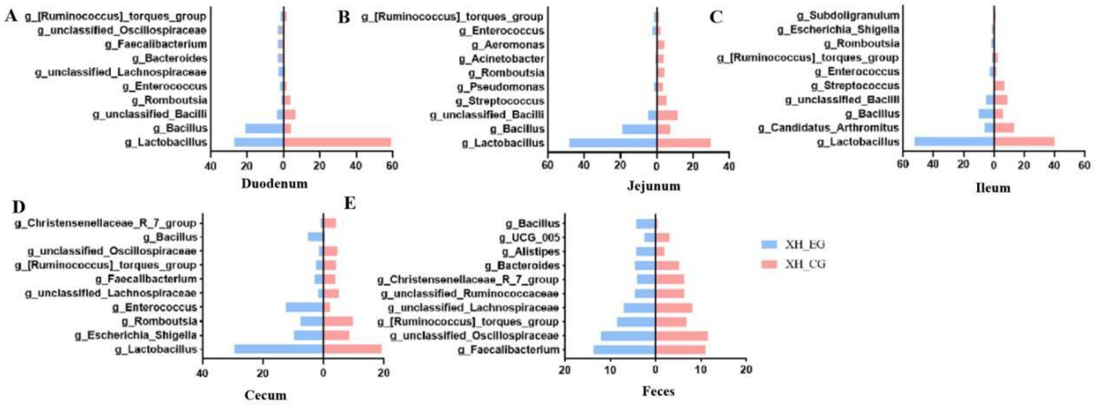

Figure S4. Effects of mixed feeding on the differential gut microbiota of 8-week-old Xinghua chickens at the genus level. The microbial differences at the genus level was shown in the duodenum (A), jejunum (B), ileum (C), cecum (D), and feces (E), respectively. XH_CG represents the control group of Xinghua chickens, and XH_EG represents the experimental group of Xinghua chickens.

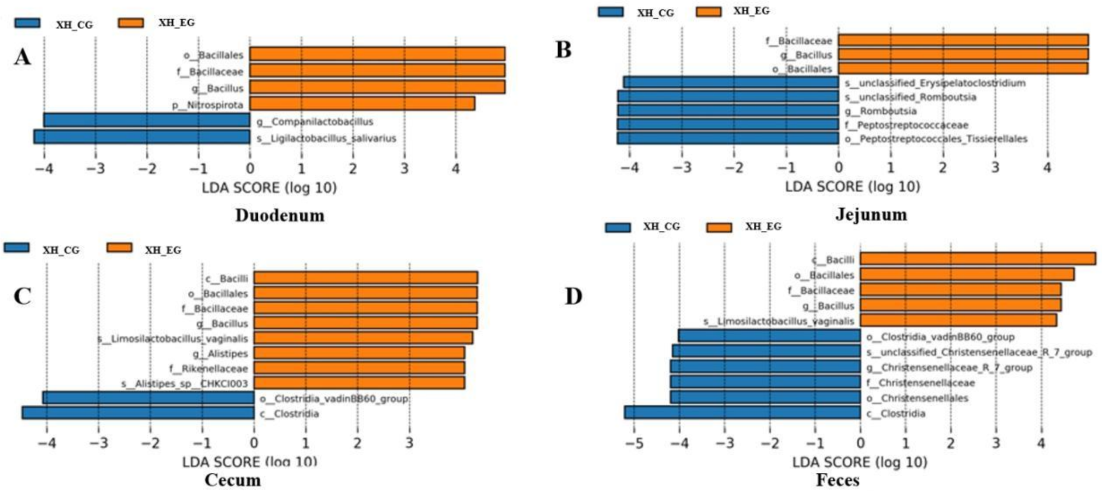

Figure S5. LDA scores of differential gut microbiota in 8-week-old Xinghua chickens. The microbial differences was shown in the duodenum (A), jejunum (B), cecum (C), and feces (D), respectively. XH_CG represents the control group of Xinghua chickens, and XH_EG represents the experimental group of Xinghua chickens.

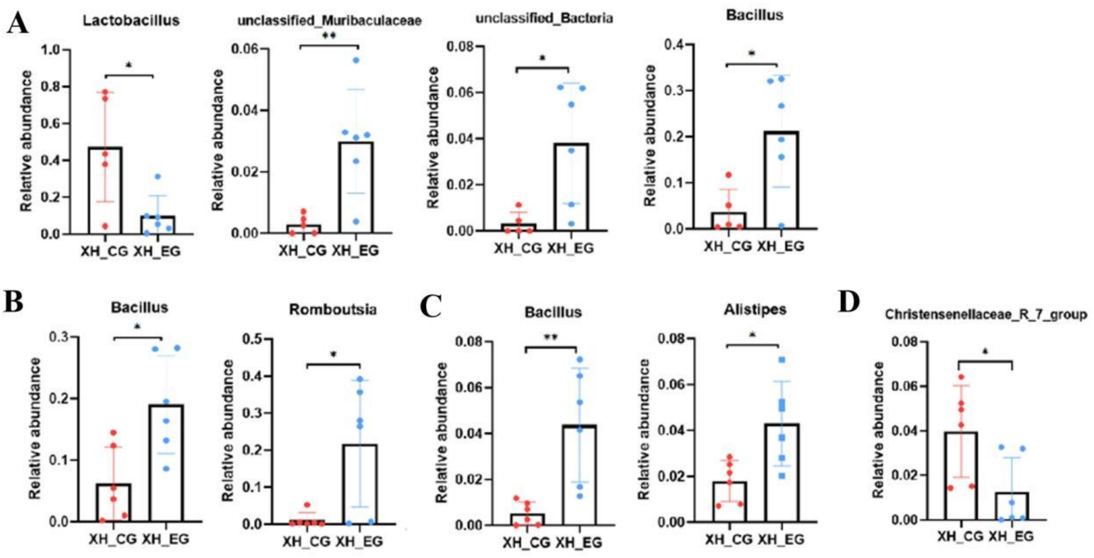

Figure S6. Partial differential genera between the two groups of Xinghua chickens. (A) Partial differential genera in the duodenum, (B) Partial differential genera in the jejunum, (C) Partial differential genera in the ileum, (D) Partial differential genera in the cecum, (E) Partial differential genera in the feces. *indicates P < 0.05; ** indicates P < 0.01; *** indicates P < 0.001.

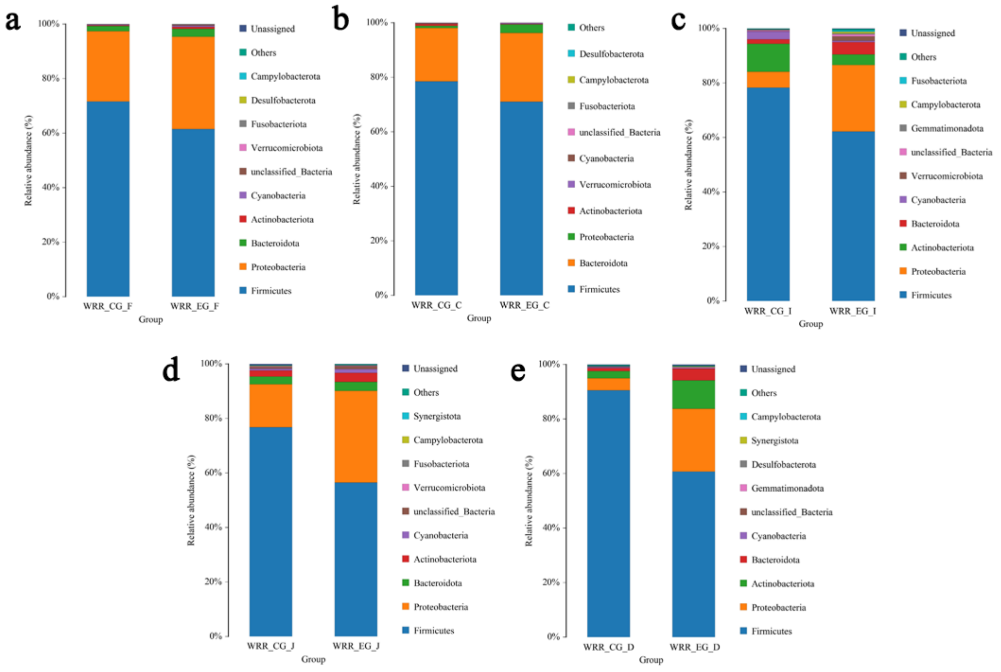

Figure S7. Effects of mixed feeding on the differential gut microbiota of 5-week-old White Recessive Rock chickens at the phylum level. The microbial

differences at the phylum level was shown in the feces (A), cecum (B), ileum (C), jejunum (D), and duodenum (E), respectively. WRR_CG represents the control group of White Recessive Rock chickens, and WRR_EG represents the experimental group of White Recessive Rock chickens.

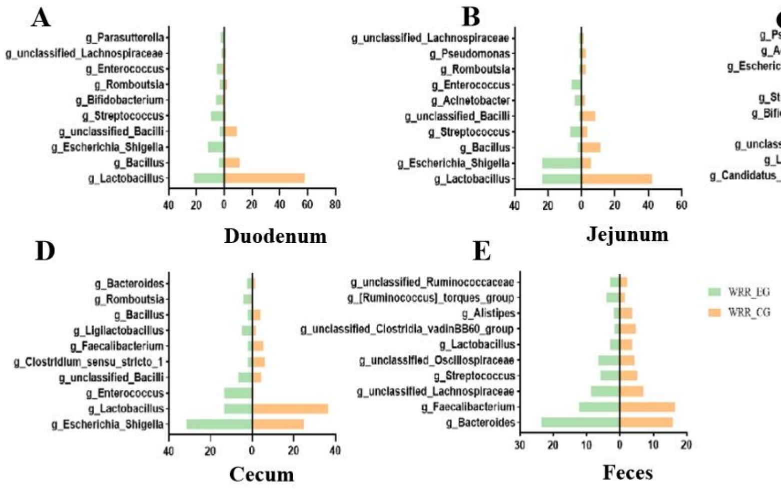

Figure S8. Effects of mixed feeding on the differential gut microbiota of 5-week-old White Recessive Rock chickens at the genus level. The microbial differences at the genus level was shown in the duodenum (A), jejunum (B), ileum (C), cecum (D), and feces (E), respectively. WRR_CG represents the control group of White Recessive Rock chickens, and WRR_EG represents the experimental group of White Recessive Rock chickens.

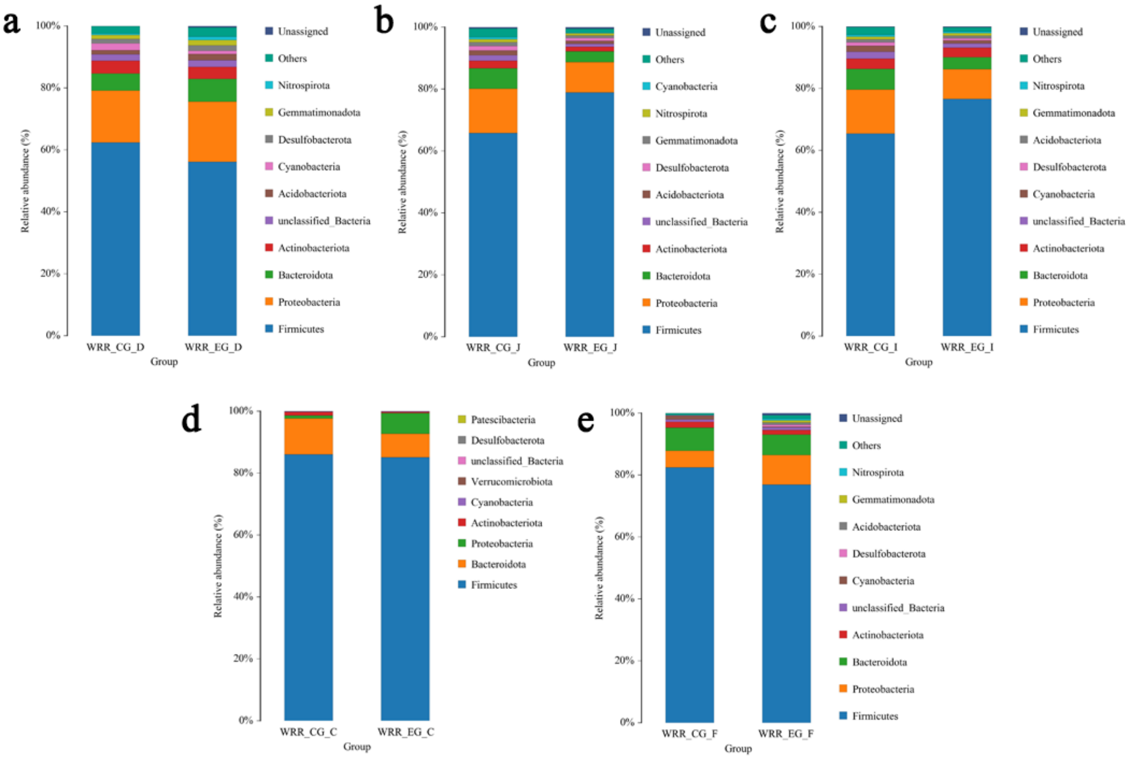

Figure S9. Effects of mixed feeding on the differential gut microbiota of 8-week-old White Recessive Rock chickens at the phylum level. The microbial differences at the phylum level was shown in the duodenum (A), jejunum (B), ileum (C), cecum (D), and feces (E), respectively. WRR_CG represents the control group of White Recessive Rock chickens, and WRR_EG represents the experimental group of White Recessive Rock chickens.
